# Early motor function after local treatment of brain metastases in the motor cortex region with stereotactic radiotherapy/radiosurgery or microsurgical resection: a retrospective study of two consecutive cohorts

**DOI:** 10.1186/s13014-017-0917-6

**Published:** 2017-11-13

**Authors:** Bogdan Pintea, Brigitta Baumert, Thomas Mehari Kinfe, Konstantinos Gousias, Yaroslav Parpaley, Jan Patrick Boström

**Affiliations:** 1 Department of Neurosurgery, University Hospital of Bonn, Sigmund-Freud-Strasse 25, 53127 Bonn, Germany; 2 Department of Neurosurgery, BG University Hospital Bergmannsheil, Bürkle-de-la-Camp-Platz 1, 44789 Bochum, Germany; 3 Department of Radiosurgery and Stereotactic Radiotherapy, MediClin Robert Janker Clinic and MediClin MVZ Bonn, Villenstrasse 8, 53129 Bonn, Germany; 4 Department of Neurosurgery, Knappschaftskrankenhaus Bochum, Ruhr University Bochum, In der Schornau 23-25, 44892 Bochum, Germany

**Keywords:** Central cerebral metastasis, Rolandic cortex, Functional outcome, Microsurgery, Radiosurgery, Stereotactic radiotherapy

## Abstract

**Background:**

We compared the functional outcome and influential factors of two standard treatment modalities for central cerebral metastases: electrophysiological-controlled microsurgical resection (MSR) and stereotactic radiotherapy/stereotactic radiosurgery (SRT/SRS).

**Methods:**

We performed a database search for central metastasis treatments during the period from January 2008 to September 2012 in two clinical registers: 1) register for intraoperative neuromonitoring (Department of Neurosurgery), and 2) prospective database for SRT/SRS (Department of Radiotherapy). Neurological status before and after treatment, Karnofsky performance index (KPI), histology, tumor localization and volume, and oncological status were standardized and pooled together for analysis. Muscle strength was graded on a scale of 0–5.

**Results:**

We identified 27 MSR and 41 SRT/SRS cases from 68 treatments. The MSR-treated patients had significant less muscle strength in the upper and lower extremities before and after the treatment as compared to the patients receiving SRT/SRS. Muscle strength of the extremities did not change for patients receiving SRT/SRS, while MSR patients had significant improvement in lower extremity muscle strength (*p* = 0.05) and a non-significant improvement in the upper extremities. MSR showed significant improvement in hemiparesis as compared to radiotherapy, but this was accompanied with a significant deterioration of extremity muscle strength after surgery, as compared to SRT/SRS (improvement *p* = 0.04, deterioration *p* = 0.10).

**Conclusion:**

Electrophysiologically guided microsurgery of central metastases had a significantly better functional outcome regarding hemiparesis. However, there was also a trend for less secondary neurological deterioration after SRT/SRS.

**Trial registration:**

ISRCTN81776764. Retrospectively Registered 27 July 2017.

## Background

Cerebral metastases are an upcoming challenge for the oncological community as the life expectancy of brain cancer patients improves and, as a consequence, the possibility of cerebral metastasis increases [1–3]. Moreover, neurological integrity and functional status is becoming an important outcome parameter, as brain cancer develops into a more chronic disease [4–6]. The mainstays of brain metastases treatment are microsurgical resection or different radiotherapy modalities, as most systemic therapeutics are efficacious treatments for cerebral metastases.

Randomized trials have demonstrated that surgical resection or radiosurgery of cerebral metastases (single or limited number), followed by postoperative whole brain irradiation (WBI) results in a mean progression-free survival of 4.6-months over each modality alone and a significant reduction by 30% in local recurrence rates [7]. However, WBI did not influence overall survival or survival with functional independence [7, 8]. In addition, WBI had an unfavorable outcome with long-term quality of life, as well as on the neurological and especially cognitive functions of patients. Therefore, new radiotherapy modalities like radiosurgery and stereotactic radiotherapy have been adopted, with both sharing a restriction of treatment to only the tumor site, which results in no prophylactic effect for any new brain metastases [7].

Microsurgical resection (MSR) and stereotactic radiotherapy/radiosurgery (SRT/SRS) become thereafter two competitive modalities in the treatment of cerebral metastasis with distinct advantages. The stereotactic radiotherapy/radiosurgery was advocated for the treatment of oligometastatic cases (≤ 3) and/or deeply situated metastases because of its less invasive character [9] and microsurgery was suggested for single and more superficially situated metastases to reduce local mass effect with faster tumor edema control [10].

Both modalities attempt to protect or improve the functionality/neurological integrity, for microsurgery by reducing the mass effect and edema in the affected but non-malignant region of the brain, as well as stereotactic radiotherapy by reducing normal tissue radiation dose. The Rolandic, or central/motor cortex area, is a suitable site to investigate the advantages and disadvantages in regards to functional outcome from microsurgery or radiation treatments, where outcomes can be assessed with standard clinical rating scales like the British Medical Research Council (BMRC) scale for motor disability [11, 12].

Some studies have revealed that both microsurgery [13, 14] and radiosurgery/stereotactic radiotherapy [15] represent a feasible option to treat lesions in the central/motor cortex areas (see Table 1). However, no previous study has directly compared these two modalities to define an optimal treatment regime, in regards to functional outcomes.

**Table 1:** Overview of published results of functional outcome of different treatment modalities (MRS vs. SRS/SRT) of brain metastases in the motor cortex region

	Patients	Tumor volume	Preoperative hemiparesis	RPA Score mean	Motoric improvement	Motoric deterioration
Weil RJ et Louser RR ^a^	17	10.2 cm^3^ (mean)	100%	1,92	88%	12%
Obermueller T et al. ^a^	56	–	57%	2	31%	21%
present series MRS ^a^	27	10.5 cm^3^ (mean)	89%	1.85	54%	11%
present series SRT/SRS ^b^	41	4.9 cm^3^ (mean)	20%	1.81	17%	5%
Luther N. et al. ^b^	96	27 pat. >9 cm^3^	51%	–	31%	37%
		69 pat. <9 cm^3^		–		12%

MRS series are marked with ^a^, SRT/SRS series are marked with ^b^

In this study, we retrospectively collected prospective data from patients receiving either microsurgery or stereotactic radiotherapy/radiosurgery from two cooperating regional treatment centers. Eligible patients were identified by searching for central metastasis treatments in a microsurgical register for intraoperative neuromonitoring and in a prospective database for stereotactic radiotherapy/radiosurgery. Both groups were standardized and pooled together for analysis. Potential factors influencing the primary endpoint of “early functional outcome,” such as neurological status before treatment, Karnofsky performance index (KPI) [16], recursive portioning analysis (RPA) score [17–19], histology, tumor localization, and tumor volume were statistically analyzed.

## Methods

### Patient populations

**Fig. 1:**
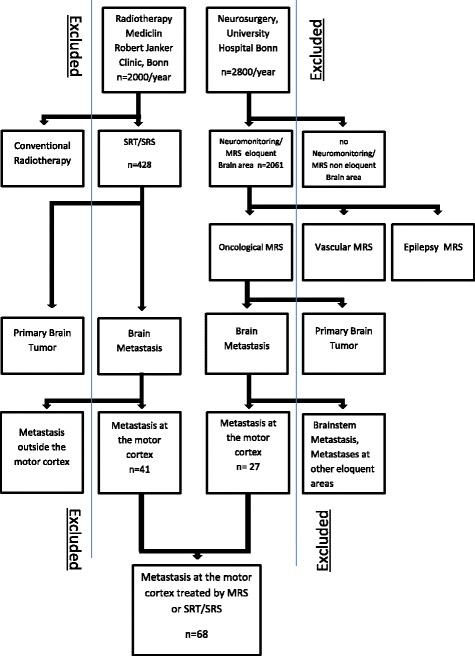
Flowchart representing the structure of our selection process for the study collective

Two prospectively documented institutional databases, 1) the register for intraoperative neuromonitoring of the Department for Neurosurgery, University of Bonn Medical Center, and 2) the database for SRT/SRS of the Department for Radiotherapy, the MediClin Robert Janker Clinic (Bonn, Germany), were retrospectively searched for cases of centrally-located cerebral metastases, details of the selection process are presented in the flow chart (Fig. 1).

We performed a retrospective analysis of 2061 cases of micro-surgically operated patients under electrophysiological control, as well as 428 cases of stereotactic radiotherapy patients during the period of January 2008 to September 2012. The particular form of treatment was decided in a weekly multidisciplinary neuro-oncology tumor board with permanent participation of senior consultants from: the neurosurgery, radiotherapy, neuroradiology, neurooncology/neurology and was conformed to current guidlines for the treatment of cerebral metastases and the latest scientific evidence. In the case of centrally located metastases, the prognosis was initially estimated based on known scores (for example, SIR: [20, 21], and the less invasive option of stereotactic irradiation was generally used primarily in patients with a survival prognosis of less than half a year. Stereotactic biopsies were not used in this series and in generally only exceptionally in suspected metastases. In the case of an unclear primary or a missing histology, a resection was recommended for histological evaluation. Otherwise the patients were offered both therapy options. After clarification on the advantages and disadvantages of both treatment options, the patients could usually choose which therapy they prefer.

For metastases with a volume of ≤4.5 cm^3^ and a 10 Gy volume of ≤10 cm^3^, a classic one session radiosurgery with a marginal dose of 20 Gy was used. In case of metastases >4.5 cm^3^ or a 10 Gy volume > 10 cm^3^, the hypofractionated variant was used (see Table 2).

**Table 2:** Overview of the applied radiation modalities, doses and fractions in SRS/SRT in relation to the tumor volume

Volume	Patients	Percentage of SRS treatments	Mean SRS dose (Gy)	Fractions(mean)	Mean SRT dose (Gy)
< 4.5 cm^3^	28	43%	20Gy	10.5	35Gy
> 4.5 cm^3^	13	7%	20Gy	10.3	33Gy

MRS was performed over a free craniotomy under general anesthesia with intravenous analgosedation. Intraoperative electrophysiology was used to identify the senso-motoric cortex. MRS was then monitored by somatosensory evoked potential and motoric evoked potentials.

### Inclusion/exclusion criteria

Cerebral metastases located within either the precentral gyrus of the motor cortex, or adjacent to the precentral gyrus in the fronto-dorsal area or the postcentral area, were included for analysis. The motor strip was identified on magnetic resonance imaging (MRI) by using standard anatomical cues, including presence of an “omega sign” in the central sulcus, termination of the superior frontal sulcus into the prefrontal sulcus, and the paracentral lobule located directly anterior to the pars marginalis. In addition, intraoperative and electrophysiologically identified cases using the phase reversal technique were included [22, 23].

Patients with no follow-up after treatment or with other cerebral metastasis that could affect the motor pathway like basal ganglia, brain stem, and spinal metastases were excluded from this study (see Fig. 1).

### Analysis of motor function

Motor function was prospectively analyzed and documented both pre- and post-procedure by each department at 1 day before and over 1–3 weeks intervals after either MSR or SRT/SRS. Motor function was graded for all patients according to the BMRC scale [11], as: normal (5/5); mild weakness (4/5); moderate weakness with the strength to overcome gravity (3/5); weakness with not enough strength to overcome gravity, but with notable movement (2/5); severe weakness with not visually but palpable movement (1/5); and absolute weakness or plegia (0/5). Hemiparesis was defined as a grade lower than 5/5 independent of the underlying reason caused by each technique (edema, hemorrhage, empyema, or collateral damage).

### Adverse events

Adverse events were retrospectively evaluated and taken in account up to 3 months after the initial therapy, by documentation in patient records or reported by practitioners afterwards. We divided the adverse events into local complications directly associated with the treatment such as bleeding, infection, radio-necrosis, and systemic adverse events not directly associated with the local treatment such as pneumonia, thrombosis, and pulmonary embolism.

### Statistical analysis

Univariate and multivariate statistical analyses were performed to determine which patient and tumor-related factors influenced functional outcome. Factors evaluated included tumor volume, patient age, tumor localization, if the tumor directly involved the motor cortex, KPI, dexamethasone daily dose, and preoperative motor deficit. We evaluated the motor function outcome in patients with and without deficits prior to each treatment. As a surrogate parameter for clinically-relevant edema, we examined dexamethasone treatment during follow-up.

These statistical comparisons were analyzed using Fisher’s exact test and Student’s t-test. Multivariate analyses were performed as binary logistic regression analysis with IBM SPSS Statistics version 20.0 (IBM Corp., North Castle, NY, USA). A *p*-value of *p* ≤ 0.05 was considered statistically significant.

## Results

From both patient databases, a total of 68 patients were identified as meeting the inclusion criteria. Of the 68 total patients, 27 were MSR treatments and 41 were SRT/SRS treatments. However, 2 SRT/SRS cases were missing data and were therefore excluded from further statistical analysis. Moreover, 1 patient had surgical resection with SRT over 3 months later, this patient included in both treatment arms. Regarding pre-operatively assessed variables, the MSR group differed significantly from the SRT/SRS group for tumor volume (10.5 cm^3^ vs. 4.9 cm^3^, *p* = 0.02), rate of preoperative hemiparesis (88.9% vs. 19.5%, *p* = 0.005), and muscle strength of the extremities (lower extremities, 4.3 vs. 4.8, *p* = 0.008; upper extremities, 3.7 vs. 4.7, *p* = 0.0005) according to the BMRC criteria. No significant difference was found between both groups regarding age, gender, primary tumor histology, pre-treatment KPI, and oncological status on the RPA score as shown in detail in Table 3.

**Table 3:** Pre-treatment demographics, oncological data, and motor status of patients with central metastasis, based on the treatment modalities, namely, MRS or SRS/SRT

	Overall	MRS	SRT/SRS	*p*
Treatments	68	27	41	
Patients (n)	64	27	37	
Females (%)	52%	52%	52%	n.s.
Age (years)	59.5	60	59	n.s.
RPA	1.83	1.85	1.81	n.s
KPI pre-operative *	78	80	78	n.s
Tumor-Volume (cm^3^) *	7.2	10.5	4.9	<0.02
Patients with Hemiparesis	36	24	12	<0.005
Muscle strength (BMRC)*:				
Arm	4.3	3.7	4.7	<0.008
Leg	4.6	4.3	4.8	<0.0005
NSCLC	43%	44.4%	42.4%	n.s
Mamma-Ca	14.5%	18.5%	11.9%	n.s.
Melanoma	13%	18.5%	9.5%	n.s.
Renal Cell Ca	5.8%	0%	9.5%	n.s.
CUP	5.8%	0%	9.5%	n.s.
SCLC	4.3%	0%	7.1%	n.s
Germ Cell Ca	4.3%	7.4%	2.4%	n.s.
Gastrointestinal Ca	4.3%	3.7%	4.8%	n.s.
Urothelial Cell Ca	2.9%	3.7%	2.4%	n.s.
Sarcoma	1.4%	3.7%	0%	n.s.

*SRT/SRS* Stereotactic radiotherapy or radiosurgery, *MRS* Microsurgery, *KPI* Karnofsky performance index, *RPA* Recursive partitioning analysis, *BMRC* British Medical Research Council, *NSCLC* Non-small cell lung cancer, *SCLC* small cell lung cancer, *Ca* Carcinoma, *CUP*: Carcinoma of unknown primary, *n.s* not significant

* mean value

When comparing the early postoperative mean muscle strength of the extremities between the MSR and SRT/SRS groups, there was no significant difference in either the upper or lower extremities. However, when considering the rate of improvement, the strength of the lower extremities improved significantly more in the MSR group (by 0.4 points), as compared to no improvement in the SRT/SRS group. Although the improvement of the upper extremities muscle strength was not significant, the MSR patients’ strength improved in the upper extremities by 0.4 points as compared to the SRT/SRS group. These details are described in Table 4.

**Table 4:** Motor outcomes and complications in patients with central metastasis based on the treatment modalities, namely, MRS vs SRT/SRS

	Overall	MRS	SRT/SRS	*p*
Muscle strength (BMRC)*				
Arm	4.5	4.1	4.7	n.s.
Leg	4.6	4.4	4.8	n.s.
Patients with motor improvements (%)	44.8%	54.2%	16.7%	<0.042
Motoric Improvement (BMRC)*				
Arm		0.5	0.1	n.s.
Leg		0.4	0.0	<0.05
Motoric Deterioration	10.7%	11.1%	5.1%	0.1
Dexamethasone daily dose (mg)		4.5	9.5	<0.001
Overall complications	31.9%	25.9%	35.7%	n.s.
Local	13%	18.5%	9.5%	n.s.
Systemic	18.8%	7.4%	26.2%	<0.06

*SRT/SRS* Stereotactic radiotherapy or radiosurgery, *MRS* Microsurgery, *BMRC* British Medical Research Council, *n.s* not significant

* mean value

An overall improvement of a pre-treatment motor deficit in the upper or lower extremity was found significantly more often in MSR patients (54% vs. 17%, *p* = 0.04); this finding was also significant in the multivariate analysis (*p* = 0.05). A localization of the metastasis adjacent to the precentral gyrus revealed a statistical trend to better improvement of the motor deficit only in the multivariate analysis (*p* = 0.067), but was not significant in the univariate analysis (Tables 4 and 5).

**Table 5:** Multivariate analysis of different influencing factors (in the first column), with the motor outcomes and complications (in the first row)

	Overall complication rate	Systemic complication rate	Local complication rate	Motoric improvement	Motoric deterioration	Dexamethasone daily doses
MRS vs SRS/SRT	–	*p* < 0.02 (correlates with SRS/SRT)	–	*p* < 0.05 (correlates with MRS)	–	*p* < 0.04 (correlates with SRS/SRT)
Age	–	*p*~0.05 * (correlates with higher age)	–	–	–	–
Tumor Volume	–	–	*p* < 0.03 (correlates with higher volume)	–	*p* < 0.01 (correlates with higher volume)	–
KPI	–	–	–	–	*p* < 0.01 (correlates with lower KPI)	–
Located at the precentral gyrus vs adjacent to precentral gyrus	–	–	–	*p* < 0.07 (correlates with location adjacent to precentral gyrus)	–	–
Dexamethasone daily doses	–	–	–	–	–	X

* a *p* value that was not constant in the different regression analysis models and x mark a factor of influence which was not taken in account for the regression analysis of this certain outcome. Non-significant *p*-values were marked with (−)

*SRT/SRS* Stereotactic radiotherapy or radiosurgery, *MRS* Microsurgery, *KPI* Karnofsky performance index

A statistical trend of a lower rate of motor function deterioration was observed in the SRT/SRS group (4.8% vs. 11.1%, *p* = 0.10). However, this trend could not be confirmed in the multivariate analysis where deterioration of the motor function showed a significant correlation only with tumor volume (*p* = 0.009) and preoperative KPI (*p* = 0.006) (Table 5).

The overall incidence of complication was 25% in the MSR group and 35.7% in the SRT/SRS group, but this difference was not statistically significant (see Table 6). However, when examining specific sites of complication (local vs. systemic), there was a higher but non-significant rate difference of local complications in the MSR group vs. SRT/SRS group (18.5% vs. 9.5%). This was confirmed in binominal regression analysis, where the only determining factor of local complication incidence was tumor volume (*p* = 0.025) (Tables 4 and 5).

**Table 6:** Overview of local and systemic complications depending the treatment modality

	Overall	MRS	SRT/SRS
Local complications:			
brain abscess and meningitis	2	2	–
seizure	3	1	2
bleeding	1	1	–
wound healing complications	1	1	–
vigilance disturbance	1	–	1
vertigo	1	–	1
Systemic complications			
pneumonia	5	1	4
lung embolism	2	1	1
candida esophagitis, gastritis	4	–	4
psychosis	2	–	2

In the univariate and multivariate analyses of systemic complications, there was a correlation with SRT/SRS (26.2% vs. 7.4%, univariate *p* = 0.06; multivariate *p* = 0.017), but only in some multivariate statistical tests with a higher patient age (Tables 4 and 5).

In the SRT/SRS group, the daily dexamethasone dose was significantly higher compared to the MSR group (9.5 mg vs. 4.5 mg, *p* = 0.001); this difference remained significant even if tumor volume, age, KPI, and tumor localization was adjusted (Tables 4 and 5).

## Discussion

### Advantages and disadvantages of microsurgery

For treatment with MSR, there seems to be a significant difference regarding the recovery of motor deficits caused by central metastases as compared with SRT/SRS. However, this recovery may also depend on other factors, such as the exact neuroanatomical localization of the metastases. From the standpoint of neurosurgery, it certainly impacts recovery if a metastasis is located either directly in the precentral gyrus or only adjacent to it. In our study, a localization of the metastasis adjacent to the precentral gyrus revealed only a statistical trend toward better improvement of the motor deficit in the multivariate analysis, but not in the univariate analysis.

The electrophysiologically controlled, intra-operative identification of the pyramidal tract is standard practice and was carried out consistently in our study. The pre-operative identification of the pyramidal tract by navigated transcranial magnetic stimulation (nTMS) [24], is increasingly utilized over the last 5 years, which implies that this technique was not available during the treatment period of our study (2008–2012). If such pre-operative methods can demonstrate that a metastasis grows directly in the pyramidal tract, such critical cases could be excluded early from operation. Alternatively, a calculated hemiparesis risk could be discussed with the patient. Moreover, this could show a reduced quality of life and therefore be declined as a treatment for these patients. These critical patients could then choose SRT/SRS as an alternative treatment.

Due to the invasive nature of MSR, our results demonstrate a higher rate of local complications such as bleeding, infection, or infarction. We believe that the precise preoperative selection of patients with a disposition for local complications is a crucial factor to improve surgical outcomes. This screening should include the exclusion of adverse blood clotting and a comprehensive evaluation of the patient’s immune system, which is supported by the neurosurgical literature [25–27]. The reduction of the local complication rate remains vital, as the start of most adjuvant therapy depends on rapid and complete wound healing and recovery post-surgery. In addition, by further improving the MSR technique and intraoperative monitoring, microsurgery should improve in both general and motor deficit outcomes.

A topic we do not assess in our study is local tumor recurrence for each treatment option. Conform to the guidelines for cerebral metastases for all patients a postoperative adjuvant radiation therapy was recommended and performed depending on the postoperative KPI. However, post-op radiation therapy was usually performed with a delay of 2–4 weeks after MSR and we did not assess these data because we focused on the early functional outcome. Recent publications cautioned not to underestimate local recurrence after microsurgery, especially if a supramarginal resection cannot be administered [28, 29]. This is particularly important in the critical central region, since resection must be conservative in this area of the brain. Microsurgery of cerebral metastases in the Rolandic area may therefore be inferior to local radiotherapy, regarding local tumor recurrence. This inferiority of MSR in comparison to SRT/SRS without adjuvant therapy has been previously described [7]. By future improvements in adjuvant systemic therapy, the progression-free survival in these cases may be less dependent on whether local therapy utilizes radiotherapy or microsurgery.

### Advantages and disadvantages of radiotherapy

Due to early detection by routine MRI staging, as well as the MRI screening during follow-up, the majority of central metastases may be small and asymptomatic upon discovery. Especially for these types of metastases, SRT/SRS seems to be the optimal choice according to our data. However, treatment of central metastases by SRT/SRS also has some disadvantages. First, SRT/SRS failed to improve motor function in patients with pre-existing motor deficits in our study. Second, and possibly related to the first, SRT/SRS has a significantly higher dose and longer duration of edema therapy, as revealed by administration of dexamethasone (SRT/SRS vs. MSR = 9.5 mg vs. 4.5 mg, *p* = 0.001). The higher and prolonged dexamethasone therapy in SRT/SRS patients may have implications beyond local impact by also having a negative systemic influence. Dexamethasone was individually administered and adapted to the neurological symptomatics (in particular the motoric function) of the patients. The highest daily dose of 24 mg was reduced regularly for about 1–2 weeks. However, the more intense dexamethasone therapy in SRT/SRS patients may be one explanation for the positive correlation between the higher rate of systemic complications with SRT/SRS observed in our study. Therefore, an improvement in edema therapy by reducing dexamethasone dose and the adoption of new molecular anti-edematous treatment approaches, such as anti-VEGF therapy [30, 31], may improve outcomes for stereotactic radiotherapy of central metastases in the future. Another explanation for the higher prevalence of systemic complications in SRT/SRS patients could be a higher proportion of ill and/or and elderly patients than found in the MSR cohort. In our study, however, there were no significant differences concerning age and KPI, with only a weak correlation between systemic complications and age in the multivariate analysis. Another key factor for improvement in the treatment outcomes of stereotactic radiotherapy could be the optimization of radiotherapy fractionation, single fraction versus hypofractionation, to reduce the rate of radiogenic edema and necrosis. The tumor volume dependence of radio-necrosis has been proven in stereotactic radiosurgery [32]. Hypofractionated stereotactic techniques are increasingly used as an alternative option in cases with a large treatment volume, in an effort to protect the surrounding normal tissue [33].

### Statistics and Limitations

Our study had some limitations. The results of this study should be interpreted with caution, due to its retrospective design. We are aware of the differences regarding tumor size and motor deficits before treatment and made efforts to taken them into account in the statistical analyses. We considered only the restricted collective with motor deficits before treatment concerning the calculation of improvement of motor deficits. In addition, we controlled all our univariate results by multivariate regression analyses. Moreover, there is a patient selection bias due to the guidelines for SRS, which usually restricts treatment to tumor sizes smaller than 2.5 cm in diameter, or 4.5 cm^3^ in volume. Despite this selections bias, we consider our results a valuable contribution, since they emphasize an often overlooked outcome parameter for the treatment of cerebral metastases, which is very important for patient quality of life: the motor function and a method to quantify and evaluate this important neurological function.

### Future perspective

As cancer becomes more a chronic disease [1–3], central cerebral metastases may become more common. As a result, neurological functional outcome may gain clinical importance in the future, in addition to oncological outcomes [4–6]. New imaging techniques may improve the detection and the resolution of metastases and the surrounding structures. This is important for the neuronavigation in microsurgery and for the target volume defined for radiotherapy. Moreover, these diagnostic techniques might be able to give molecular properties, which can be taken into consideration for future treatment decisions. Furthermore, new bio-molecular drugs [30, 31] and bio-molecular materials [34], may improve the overall oncological outcomes, as well as control the side effects of both microsurgery [34–36] and stereotactic radiotherapy [37] in the future.

## Conclusions

To the best of our knowledge, this is the first report to compare the two standard treatment options, MRS and SRT/SRS, of central cerebral metastases by examining their functional outcome and risk profile. Our data showed that the presence of a pre-operative motor deficit, as well as the tumor volume, should be taken in consideration when deciding the treatment modality. However, since microsurgical resection remains an invasive therapy, evaluation of the patient’s blood clotting function and the immune system, should be considered for reducing complications in microsurgery.

## Abbreviations

BMRC: British Medical Research Council
MRI: Magnetic resonance imaging
MSR: Microsurgical resection
SRS: Stereotactic radiosurgery
SRT: Stereotactic radiotherapy
